# Acute valve malfunction with thrombosed bioprosthetic valve after surgical aortic valve replacement

**DOI:** 10.1002/ccr3.7973

**Published:** 2023-09-27

**Authors:** Hiroki Moriuchi, Masaaki Koide, Yoshifumi Kunii, Takuya Maeda, Risa Shimbori

**Affiliations:** ^1^ Department of Cardiovascular Surgery SeireiHamamatsu Hospital Hamamatsu Japan

**Keywords:** bioprosthetic aortic valve, thrombus

## Abstract

Acute valve thrombosis after bioprosthetic aortic valve replacement even under anticoagulation therapy is extremely rare. Cardiac computed tomography is a powerful imaging tool to detect valve thrombosis, and surgery is necessary in case of unstable hemodynamics.

## Graphical abstract

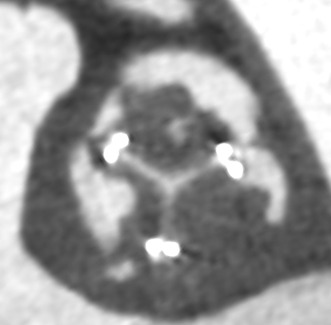

## 1 CASE PRESENTATION

A 74‐year‐old man with chest pain was diagnosed with Stanford type A acute aortic dissection and severe aortic regurgitation. Aortic valve replacement (AVR) with a 21‐mm INSPIRIS (Edwards Lifesciences) and ascending aortic replacement with 26‐mm J Graft (Japan Lifeline) were performed. The Inspiris valve was implanted using a single interrupted suture technique, and there was no injury to the valve. During cardiopulmonary bypass (CPB), the activated clotting time (ACT) values exceeded 480 s. The durations of circulatory arrest, cross clamp, and CPB were 43, 154, and 215 min, respectively. Postoperative transesophageal echocardiography (TEE) confirmed good motion of the bioprosthetic valve, and weaning from CPB was successfully accomplished using dopamine at a rate of 6 μg/kg/min and dobutamine at a rate of 6 μg/kg/min.

**FIGURE 1:**
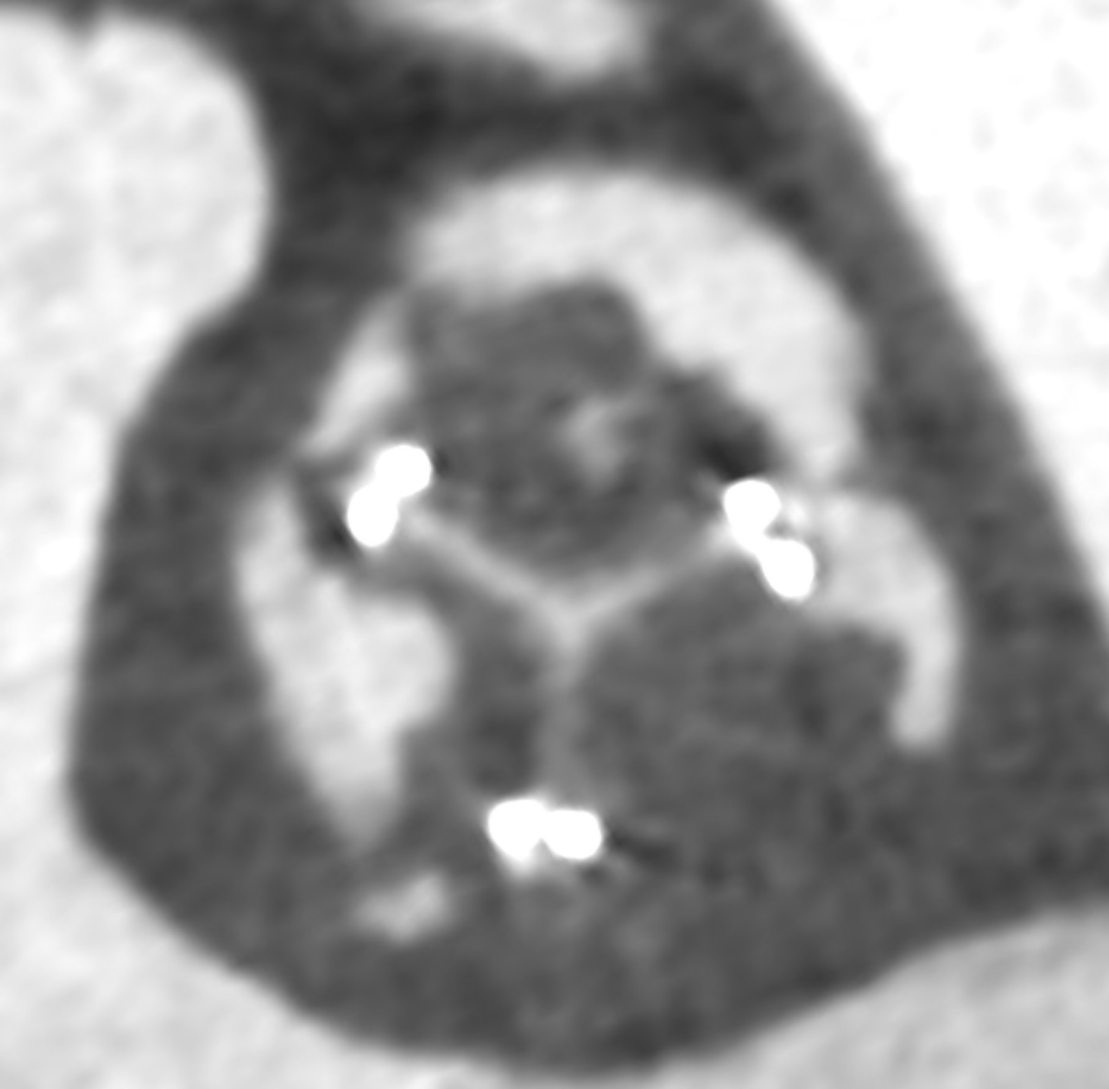
Computed tomography (CT) shows thrombi in all leaflets.

**FIGURE 2:**
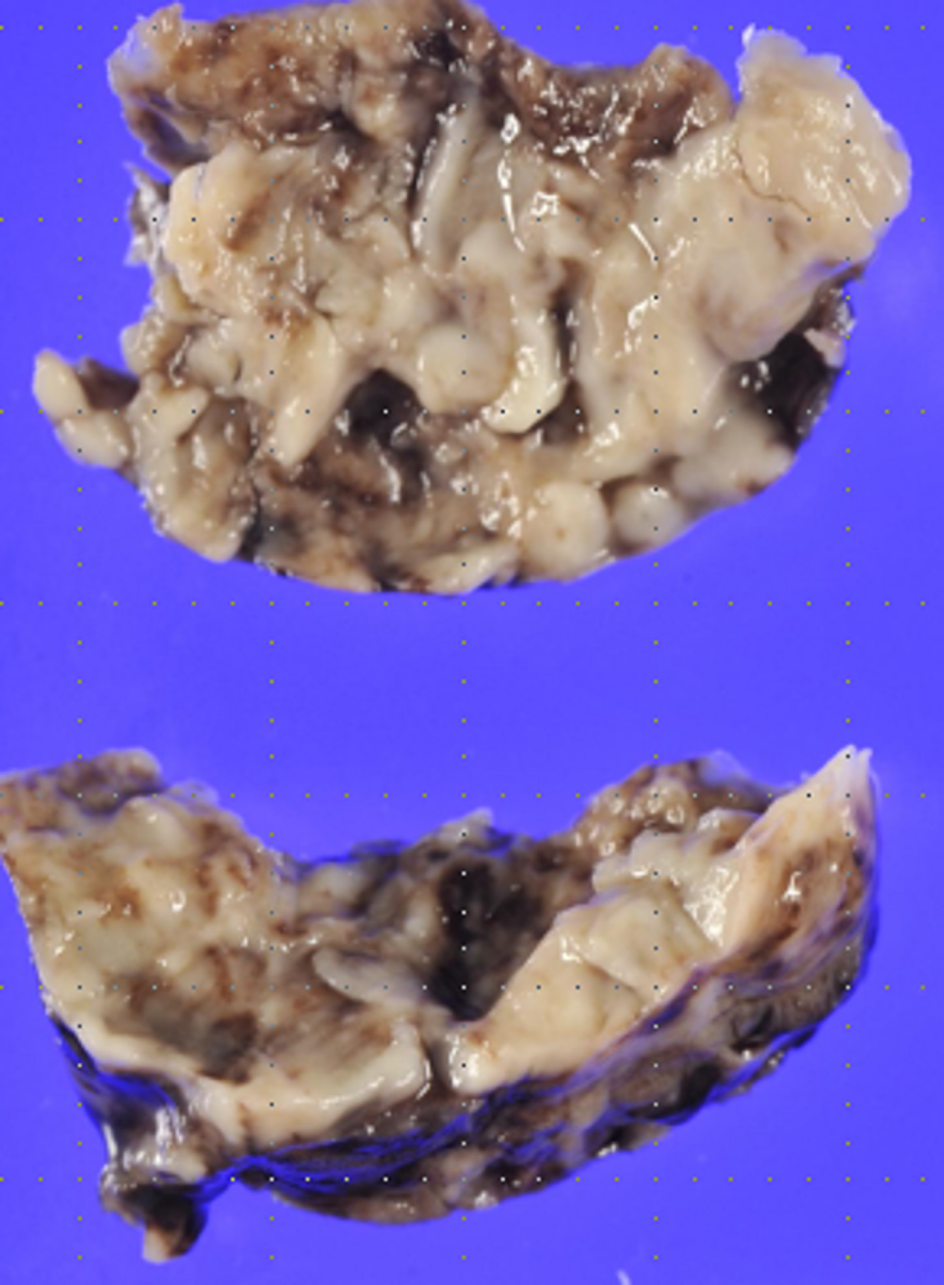
All thrombi were removed completely.

**FIGURE 3:**
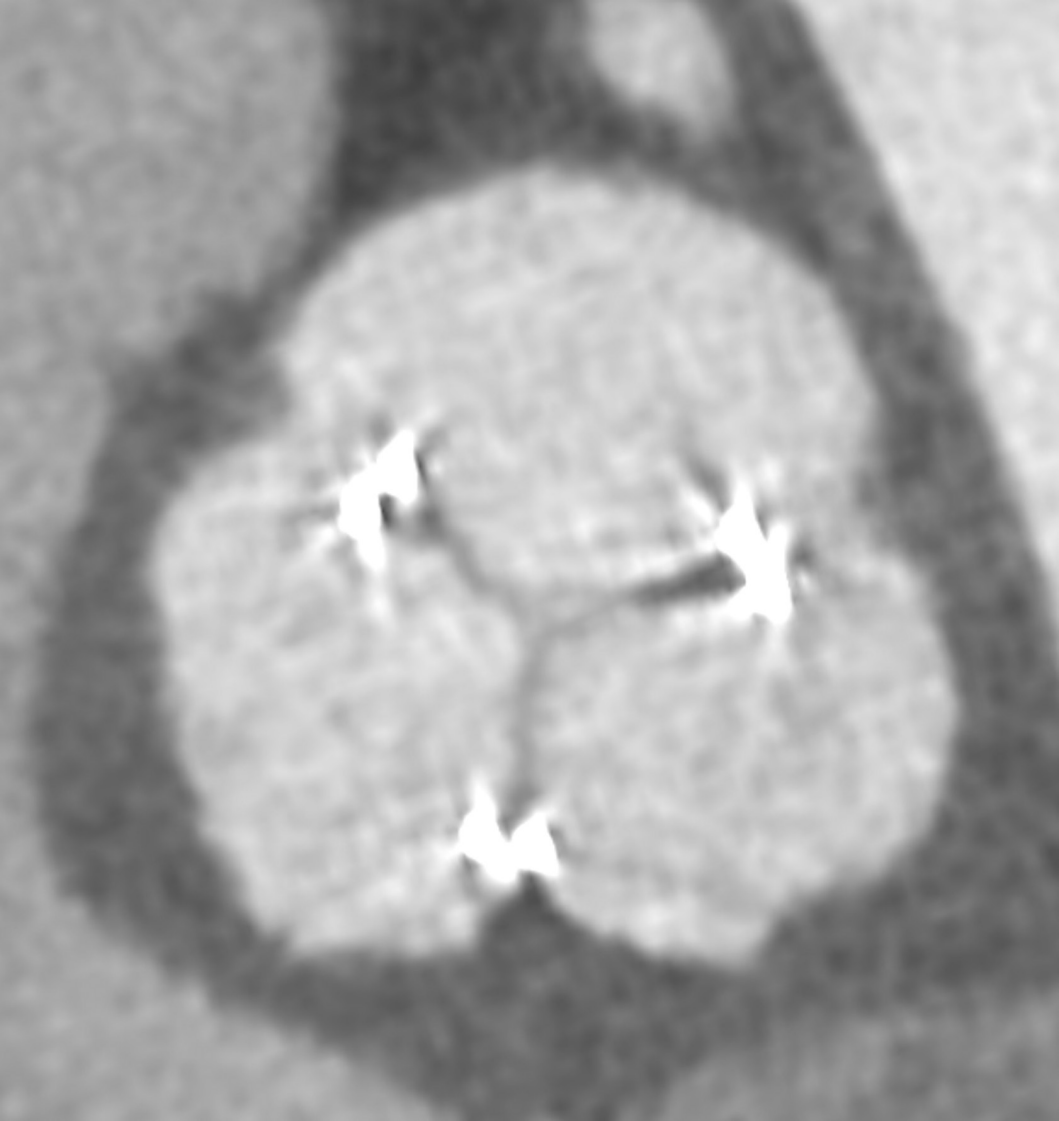
Postoperative CT shows no thrombus in bioprosthetic aortic valve.

Tranexamic acid was administered once immediately after surgery. Postoperative day (POD) 2, we initiated anticoagulation therapy using heparin and warfarin. The target ranges for activated partial thromboplastin time (APTT) and prothrombin time‐International Normalized Ratio (PT‐INR) were set at 50–60 and 1.5–2.0, respectively. Postoperative computed tomography (CT) revealed thrombus formation to all leaflets of the implanted valve (Figure 1) and echocardiography revealed restricted leaflet motion. The thrombosed biological valve demonstrated a maximum velocity of 3.2 m/sec, a mean pressure gradient of 26 mmHg, and an aortic valve area of 0.6 cm^2^. The patient's symptom of heart failure was progressive due to valve thrombosis, emergent surgery was performed on POD 10. Abundant fresh thrombi adhered to all leaflets of the bioprosthetic valve, and those thrombi were carefully removed (Figure 2). No damage or macroscopic degeneration on the leaflets was seen, so the implanted valve was preserved. Due to the potential risk of heparin‐induced thrombocytopenia (HIT), we did not use heparin in the postoperative period. We administered warfarin along with aspirin (100 mg/day) and the target INR was set at 2.5–3.0 to prevent recurrence of thrombus formation. Postoperative CT showed no sign of thrombus (Figure 3).

Bioprosthetic aortic valve thrombosis is rare complication after surgical AVR compared to transcatheter AVR. Specifically, reduced leaflet motion leading to heart failure is extremely rare.^1^, ^2^

The mechanism behind valve thrombosis is not yet fully understood, but it involves multiple factors. Hemostatic factors such as obesity, pregnancy, and inadequate anticoagulation therapy, as well as hemodynamic factors like low cardiac output, mitral or tricuspid valve issues, and patient‐prosthesis mismatch are believed to contribute to valve thrombosis. Additionally, endothelial factors such as leaflet injury during surgery and the biocompatibility of the prosthesis itself are also considered potential causes of valve thrombosis.^3^ In this case, we initiated anticoagulation with heparin and warfarin, but it is possible that this treatment regimen was not sufficiently effective for this patient. In addition, we consider perioperative cardiac low output to be the cause of valve thrombosis. Anticoagulation therapy after AVR is important and cardiac CT is a powerful tool to diagnose valve thrombosis. In cases where hemodynamic instability is present, emergent surgery becomes necessary.

## AUTHOR CONTRIBUTIONS

**Hiroki Moriuchi:** Conceptualization. **Masaaki Koide:** Writing – review and editing. **Yoshifumi Kunii:** Conceptualization. **Takuya Maeda:** Writing – review and editing. **Risa Shimbori:** Conceptualization.

## FUNDING INFORMATION

None.

## CONFLICT OF INTEREST STATEMENT

The authors report no conflict of interest.

## CONSENT

Written informed consent was obtained from the patient to publish this report in accordance with the journal's patient consent policy.

## Data Availability

None.
